# Radiation enhancement using focussed ultrasound-stimulated microbubbles for breast cancer: A Phase 1 clinical trial

**DOI:** 10.1371/journal.pmed.1004408

**Published:** 2024-05-17

**Authors:** Daniel Moore-Palhares, Archya Dasgupta, Murtuza Saifuddin, Maria Lourdes Anzola Pena, Shopnil Prasla, Ling Ho, Lin Lu, Joseph Kung, Evan McNabb, Lakshmanan Sannachi, Danny Vesprini, Hanbo Chen, Irene Karam, Hany Soliman, Ewa Szumacher, Edward Chow, Sonal Gandhi, Maureen Trudeau, Belinda Curpen, Greg J. Stanisz, Michael Kolios, Gregory J. Czarnota

**Affiliations:** 1 Department of Radiation Oncology, Sunnybrook Health Sciences Centre, Toronto, Canada; 2 Department of Radiation Oncology, University of Toronto, Toronto, Canada; 3 Physical Sciences, Sunnybrook Research Institute, Toronto, Canada; 4 Division of Medical Oncology, Sunnybrook Health Sciences Centre, Toronto, Canada; 5 Department of Medicine, University of Toronto, Toronto, Canada; 6 Department of Medical Imaging, Sunnybrook Health Sciences, Toronto, Canada; 7 Department of Medical Imaging, University of Toronto, Toronto, Canada; 8 Department of Biophysics, University of Toronto, Toronto, Canada; 9 Department of Neurosurgery, Medical University, Lublin, Poland; 10 Toronto Metropolitan University, Toronto, Canada; Washington University in St Louis, UNITED STATES

## Abstract

**Background:**

Preclinical studies have demonstrated that tumour cell death can be enhanced 10- to 40-fold when radiotherapy is combined with focussed ultrasound-stimulated microbubble (FUS-MB) treatment. The acoustic exposure of microbubbles (intravascular gas microspheres) within the target volume causes bubble cavitation, which induces perturbation of tumour vasculature and activates endothelial cell apoptotic pathways responsible for the ablative effect of stereotactic body radiotherapy. Subsequent irradiation of a microbubble-sensitised tumour causes rapid increased tumour death. The study here presents the mature safety and efficacy outcomes of magnetic resonance (MR)-guided FUS-MB (MRgFUS-MB) treatment, a radioenhancement therapy for breast cancer.

**Methods and findings:**

This prospective, single-center, single-arm Phase 1 clinical trial included patients with stages I–IV breast cancer with in situ tumours for whom breast or chest wall radiotherapy was deemed adequate by a multidisciplinary team (clinicaltrials.gov identifier: NCT04431674). Patients were excluded if they had contraindications for contrast-enhanced MR or microbubble administration. Patients underwent 2 to 3 MRgFUS-MB treatments throughout radiotherapy. An MR-coupled focussed ultrasound device operating at 800 kHz and 570 kPa peak negative pressure was used to sonicate intravenously administrated microbubbles within the MR-guided target volume. The primary outcome was acute toxicity per Common Terminology Criteria for Adverse Events (CTCAE) v5.0. Secondary outcomes were tumour response at 3 months and local control (LC). A total of 21 female patients presenting with 23 primary breast tumours were enrolled and allocated to intervention between August/2020 and November/2022. Three patients subsequently withdrew consent and, therefore, 18 patients with 20 tumours were included in the safety and LC analyses. Two patients died due to progressive metastatic disease before 3 months following treatment completion and were excluded from the tumour response analysis. The prescribed radiation doses were 20 Gy/5 fractions (40%, *n* = 8/20), 30 to 35 Gy/5 fractions (35%, *n* = 7/20), 30 to 40 Gy/10 fractions (15%, *n* = 3/20), and 66 Gy/33 fractions (10%, *n* = 2/20). The median follow-up was 9 months (range, 0.3 to 29). Radiation dermatitis was the most common acute toxicity (Grade 1 in 16/20, Grade 2 in 1/20, and Grade 3 in 2/20). One patient developed grade 1 allergic reaction possibly related to microbubbles administration. At 3 months, 18 tumours were evaluated for response: 9 exhibited complete response (50%, *n* = 9/18), 6 partial response (33%, *n* = 6/18), 2 stable disease (11%, *n* = 2/18), and 1 progressive disease (6%, *n* = 1/18). Further follow-up of responses indicated that the 6-, 12-, and 24-month LC rates were 94% (95% confidence interval [CI] [84%, 100%]), 88% (95% CI [75%, 100%]), and 76% (95% CI [54%, 100%]), respectively. The study’s limitations include variable tumour sizes and dose fractionation regimens and the anticipated small sample size typical for a Phase 1 clinical trial.

**Conclusions:**

MRgFUS-MB is an innovative radioenhancement therapy associated with a safe profile, potentially promising responses, and durable LC. These results warrant validation in Phase 2 clinical trials.

**Trial registration:**

clinicaltrials.gov, identifier NCT04431674.

## Abstract 2

Daniel Moore-Palhares and team present the mature safety and efficacy outcomes of magnetic resonance-guided focussed ultrasound-stimulated microbubble (MRgFUS-MB) treatment, a radioenhancement therapy for breast cancer.

## Author summary

**Why was this study done?:**

- Preclinical studies demonstrated combining radiotherapy with focused ultrasound-stimulated microbubble treatment (FUS-MB) enhances tumour cell death by 10 to 40 times, suggesting a promising radioenhancement therapy.
- Explanation: The exposure of microbubbles (intravascular gas microspheres) to acoustic waves leads to bubble cavitation, disrupting tumour vasculature and triggering endothelial cell apoptotic pathways.
- Subsequent irradiation of a microbubble-sensitised tumour significantly increases tumour death.

**What did the researchers do and find?:**

- This Phase 1 clinical trial analysed 18 patients with stages I–IV breast cancer with in situ tumours for whom breast or chest wall radiotherapy was conducted per standard-of-care.
- Patients underwent 2 to 3 sessions of magnetic resonance (MR)-guided FUS-MB treatment during their radiotherapy regimen.
- The MR-guided FUS-MB treatment demonstrated safety and achieved high rates of objective response and sustained long-term local control.

**What do these findings mean?:**

- This is, to our knowledge, the first in-human clinical trial to demonstrate the effectiveness and safety of this radioenhancement therapy.
- This study opens new avenues for improving treatment outcomes in breast cancer and the possibility of replication in other primary malignancies.
- The safety and efficacy findings observed in this trial warrant validation in Phase 2 clinical trials.

## Introduction

Radiotherapy plays a pivotal role in breast cancer management. It is typically administered in the adjuvant setting to reduce locoregional recurrence or in a palliative scenario to alleviate symptoms such as pain or bleeding [1]. However, there has been a rising interest in utilising radiotherapy in the neoadjuvant setting or as a definitive treatment for older or frail patients who may not be suitable for surgical resection [2–6]. Data from Phase I clinical trials indicate that the pathologic complete response rate following neoadjuvant radiotherapy is under 42%, demonstrating that radiotherapy alone cannot eliminate cancer completely for most patients [2,5]. Therefore, a therapeutic opportunity exists for studying selective agents or methods that could enhance the effectiveness of radiation-induced tumour cell death when treating in situ tumours.

In recent years, significant research has been conducted into using focussed ultrasound-stimulated microbubbles to enhance vascular permeability for potential oncological applications [7–14]. Microbubbles are tiny gas-filled spheres primarily used as intravascular contrast agents in ultrasound imaging. When microbubbles are exposed to an acoustic field, bubble cavitation within the targeted area (i.e., tumour) temporarily and reversibly opens endothelial vessel walls [15–17]. This process effectively increases vasculature permeability, thereby facilitating the transportation of therapeutic agents like chemotherapy or targeted therapy [18]. Ultrasound-stimulated microbubbles have also demonstrated potential in enhancing the permeability of the blood–brain barrier, facilitating the release of tumour biomarkers into the bloodstream for liquid biopsy and improving the penetration of antineoplastic drugs into the central nervous system [10,19].

Furthermore, other recent extensive studies have revealed that the disruption of endothelial cells activates specific pro-apoptotic pathways, such as the acid sphingomyelinase (ASMase)-ceramide pathway, which are typically activated by ablative radiation doses (>8–10 Gy per fraction) [8,10]. Preclinical studies have demonstrated a synergistic interaction between radiation and ultrasound-stimulated microbubbles, resulting in a 10- to 40-fold increase in tumour cell death when these treatments are combined [8–14,20]. The rationale is that when a microbubble-sensitised tumour is subsequently treated with radiation, there is increased apoptosis of endothelial cells, a consequent reduced microvascular density, and enhanced anoxic tumour cell death. This was demonstrated, for example, by Czarnota and colleagues [14], who identified a mean tumour cell death of only 4% (± 2%) when a single fraction of 2 Gy was delivered versus 44% (± 13%) when focussed ultrasound-stimulated microbubble was combined with a 2 Gy fraction, and 70% (± 8%) when it was combined with an 8 Gy fraction.

Based on promising outcomes observed in preclinical studies [12–14,20,21], we conducted the first Phase I clinical trial to evaluate the safety and effectiveness of utilising magnetic resonance (MR)-guided focussed ultrasound-stimulated microbubble (MRgFUS-MB) treatment for breast cancer patients with in situ tumours. We previously published preliminary data on the first 8 treated patients [22] and now report the mature outcomes of this innovative radioenhancement therapy.

## Methods

### Study design and participants

This prospective Phase 1, single-center, single-arm, investigator-initiated study aimed to evaluate the safety and efficacy of combining MRgFUS-MB, an radioenhancement treatment, with any radiation dose fractionation considered appropriate for the treatment of primary or recurrent breast tumours in situ. This study aimed to enroll 20 patients referred for radiotherapy at Sunnybrook Health Sciences Centre. Eligibility criteria included patients older than 18 years with stages I–IV breast cancer who required radiation therapy to a primary or recurrent breast or chest wall tumour in situ, as determined by a multidisciplinary team of medical, surgical, and radiation oncologists. Exclusion criteria included contraindications to contrast-enhanced MR (i.e., the presence metallic implants), contraindications to microbubbles administration (i.e., prior allergic reaction or significant comorbidities such as cardiac insufficiency or chronic kidney disease), abnormal coagulation profile or liver/renal function, weight over 140 kg, index lesion with relevant ulceration or bleeding, use of anticoagulants, and an Eastern Cooperative Oncology Group (ECOG) performance status ≥3. This study adhered to good clinical practice guidelines and followed the principles outlined in the Helsinki declaration. All study participants provided a written consent form before study participation. Demographic and clinical data were collected from electronic medical records, including the patients’ age, tumour histology and laterality, hormone-receptor status, staging, previous treatments, and radiation therapy details such as prescribed dose and fractionation. The study protocol was approved by the Sunnybrook Health Sciences Centre institutional research ethics committee (#3624) and registered with clinicaltrials.gov (identifier NCT04431674) in June 2020. This study is reported as per the Transparent Reporting of Evaluations with Nonrandomized Designs (TREND) Statement (**S1 Checklist**).

### Magnetic resonance-guided focussed ultrasound platform

A focussed ultrasound device (Profound Medical/Philips Sonalleve—Profound Medical, Mississauga, Canada/Philips Healthcare, Best, the Netherlands) was integrated into an MR flat couch. Adjustments were made to output power (pressure) using an estimate of ultrasound-attenuation in soft tissue of 0.5 to 0.75 dB (MHz cm) to ensure a peak negative pressure of 570 kPa at focus, based on the preclinical experimental data [9,12–14]. These parameters along with the duty cycle used (described further below) ensure that no heating was induced, while treatment temperature was monitored in real-time using MR imaging during the procedure. The MR platform consisted of a Philips Ingenia Elition X system (Philips Healthcare, the Netherlands) equipped with a 70 cm bore and a magnetic field of 3.0T.

### Procedures

**Fig 1:**
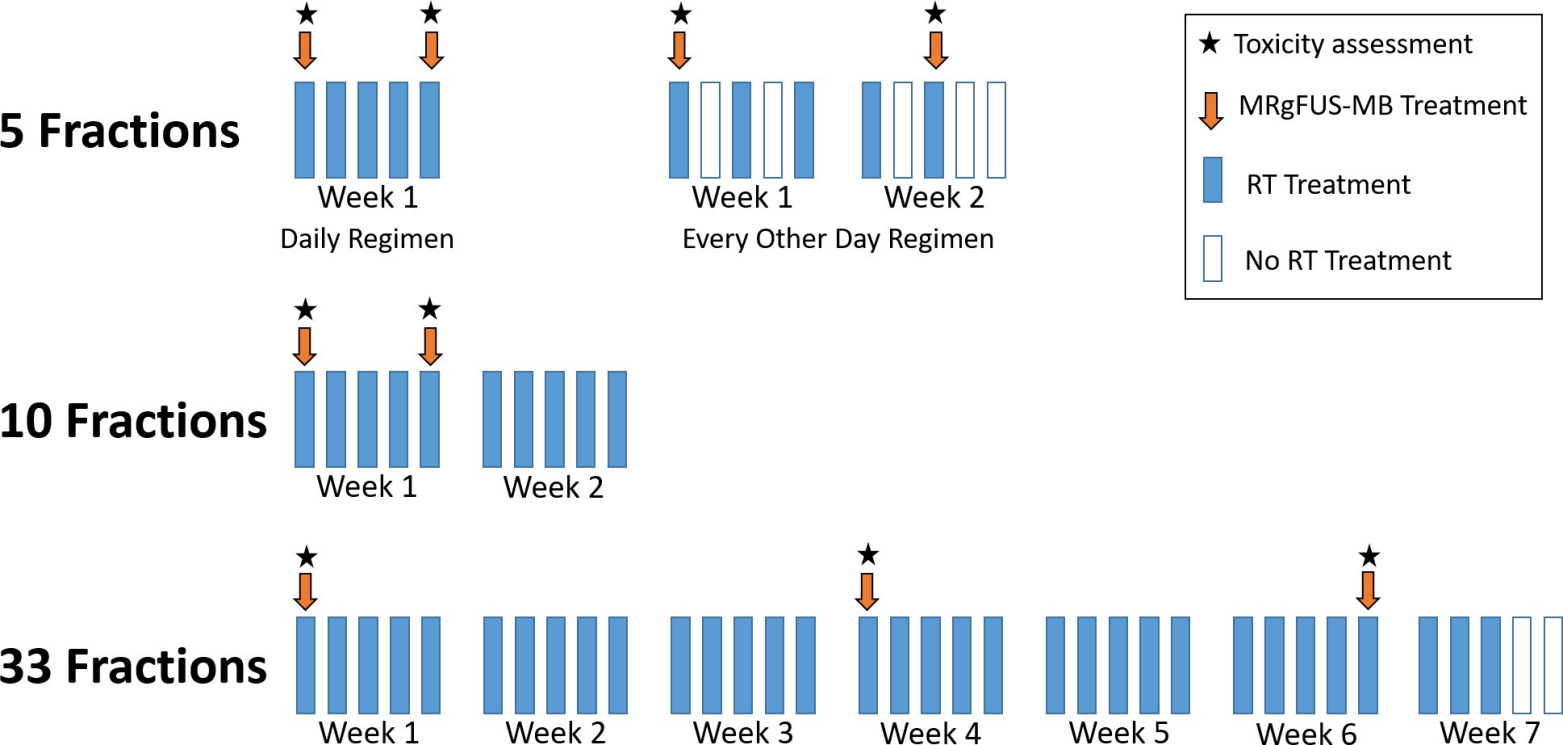
Magnetic resonance-guided focussed ultrasound-stimulated microbubble treatment schedule. MRgFUS-MB, magnetic resonance-guided focussed ultrasound-stimulated microbubble treatment; RT, radiotherapy.

Radiotherapy and MRgFUS-MB treatment were conducted on an outpatient basis. The radiation simulation process included computed tomography for all patients, with the possibility of additional MR imaging simulation [23] for enhanced contouring, determined at the discretion of the treating physician. The target tumour was delineated prospectively using all the available imaging modalities to guide treatment planning. Decisions regarding the radiation target volume and dose were made independently by the treating radiation oncologist, and any dose fractionation considered appropriate for the treatment of primary or recurrent breast tumours in situ was allowed. Radiotherapy was delivered with a computed tomography image-guided linear accelerator employing forward-planning field-in-field technique (three-dimensional conformal radiotherapy, 3D-CRT) using a static multileaf collimator, inverse-planning intensity-modulated radiotherapy (IMRT), or electrons. For patients treated with 5 to 10 radiation fractions, MRgFUS-MB was delivered before fractions 1 and 5, and for those treated with 33 fractions, before fractions 1, 16, and 30 (Fig 1).

**Fig 2:**
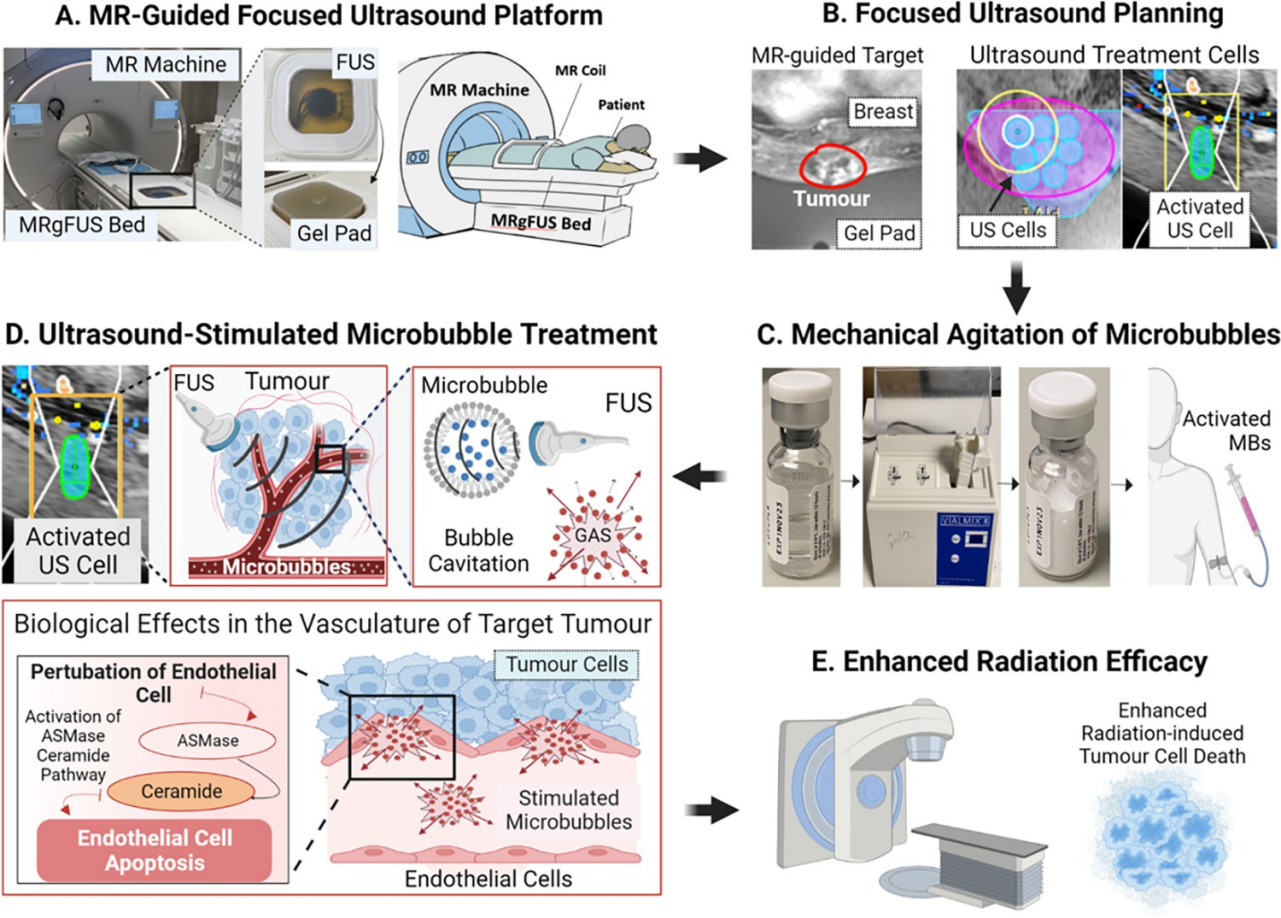
Methodology of MR-guided focussed ultrasound-stimulated microbubble treatment. **(A) MR-Guided focussed ultrasound platform.** The focussed ultrasound system was integrated into the magnetic resonance table. The patient lies in a prone position with the target tumour in contact with a gel pad positioned over the ultrasound transducer. **(B) Focussed ultrasound planning.** Magnetic resonance imaging was used by the treating radiation oncologist to delineate the target tumour (represented in red). Individual cylindrical ultrasound cells (2.8 cm in height and 1 cm in diameter) were placed in 3 dimensions over the acquired magnetic resonance images to cover the entire target tumour (represented in blue). **(C) Mechanical agitation of microbubbles.** Microbubbles (intravascular gas microspheres encapsulated by a lipid shell) are mechanically agitated using a Vialmix unit for 45 s. Subsequently, they are intravenously injected at a dose of 10–30 μl/kg, followed by a 10 ml saline flush per ultrasound cell. **(D) Ultrasound-stimulated microbubble treatment.** The ultrasound cells are sequentially activated (represented in green) until the complete target volume has been treated. Acoustic exposure of microbubbles within the target volume leads to bubble cavitation. This phenomenon induces perturbation of the tumour vasculature, leading to biomechanical effects such as increased perfusion and activation of pro-apoptotic pathways (i.e., acid sphingomyelinase [ASMase]-ceramide pathway). **(E) Enhanced radiation efficacy.** Subsequent irradiation of the microbubble-sensitised tumour results in increased apoptosis of endothelial cells, reduced microvascular density, and enhanced anoxic tumour cell death. FUS, focussed ultrasound; MR, magnetic resonance; MBs, microbubbles; MRgFUS, magnetic resonance-guided focused ultrasound; US, ultrasound, ASMase, acid sphingomyelinase.

On the days of MRgFUS-MB treatment, a peripheral intravenous line was inserted to administer MR contrast media (Gadavist; Bayer Healthcare Pharmaceuticals, Leverkusen, Germany) and microbubbles (Definity, Lantheus Medical Imaging, Billerica, Massachusetts, United States of America). Patients were positioned prone with the target tumour placed in contact with an ultrasound gel pad (Aquaflex; Parker, Hannover, Germany), which was used to minimise irregularities or gaps between the ultrasound transducer and the target. A T1-weighted MR imaging of the region of interest was acquired with the patient lying in the treatment position. The treating radiation oncologist utilised the MR images to delineate the target and position individual ultrasound treatment cells (cylindrical shape, 2.8 cm in height, and 1 cm in diameter) to cover the entire treatment volume. Consequently, the number of treatment cells varied depending on the tumour size. Definity microbubbles (Lantheus Medical) were activated by vigorous mechanical agitation on a Vialmix unit (Lantheus Medical Imaging, USA) for 45 s and injected intravenously at a dosage of 10 to 30 μl/kg followed by 10 ml saline flush for each treatment cell. Following microbubbles injection, the focussed ultrasound sequentially activated each individual cell with a precise boundary (penumbra) of ≤60 microns. A specific pulse sequence composed of a 16-cycle tone burst lasting 50 milliseconds was utilised, followed by a delay time of 1,950 milliseconds before repeating the sequence. This pattern was repeated over 5 min, resulting in a total insonication time of 750 milliseconds per treatment cell. The individual treatment cells were activated sequentially, employing a step-and-shoot approach until the entire target volume was treated. For example, in the scenario where a tumour required 4 ultrasound treatment cells, the first cell was promptly activated after intravenous microbubble administration, followed by a brief pause of 30 to 60 s for additional intravenous microbubble administration, then immediate activation of the second cell, continuing until the entire volume was treated. Following the procedure, a contrast-enhanced T1-weighted MRI scan was acquired, the patient was monitored for 30 min, and then transferred to the linear accelerator to undergo radiotherapy within 1 h of ultrasound-stimulated microbubble treatment completion. The treatment methodology was exemplified in Fig 2.

### Outcomes

The primary outcome was the incidence of acute adverse events (≤3 months after treatment completion) and was assessed for all tumours that received at least 1 MRgFUS-MB treatment. For safety purposes, the study established stopping rules to suspend the study prematurely if 6 or more of the first 10 patients experienced Grade ≥3 toxicity likely related to the intervention within 2 weeks of treatment completion, or if any serious adverse event arose that called into question the safety of the experimental treatment. Secondary outcomes included radiological response at 3 months, evaluated for participants who completed a minimum 3-month follow-up, and local control (LC).

The treating radiation oncologist assessed toxicity effects on the days of MRgFUS-MB treatment and at 1 week, 1 month, and 3 months after treatment completion. Tumour response was assessed using contrast-enhanced MR at 3 months posttreatment. Follow-up appointments beyond 3 months posttreatment were not specified in the protocol; nonetheless, they usually involved clinical assessments and computed tomography imaging every 3 to 6 months, as determined by the standard care and clinical judgement of the treating radiation/medical oncologist. The long-term radiological follow-up was used to compute data of LC.

Toxicity was graded using the Common Terminology Criteria for Adverse Events (CTCAE) version 5.0. Tumour response was evaluated according to the Response Evaluation Criteria in Solid Tumors V1.1 (RECIST) [24]. In detail, the tumour treated with MRgFUS-MB was designated as the target lesion. The sum of the largest diameter of the target lesion (contrast-enhancing lesion) was measured on baseline and follow-up MR scans, and in exceptional cases where MR data was unavailable, on CT scans. Complete response was characterised by the disappearance of the contrast-enhancing tumour on contrast-enhanced T1-weighted MR. If there was conversion of the target to residual non-enhancing tissue it was deemed replacement fibrosis (scarring) and interpreted as a complete response. Partial response was defined as a reduction of >30% in the sum of diameters of the target lesion, and progressive disease as an increase of >20% in the sum of diameters of target lesions, compared to the baseline sum diameters. Stable disease was determined if there was no significant decrease or increase in tumour size meeting the criteria for partial response or progressive disease. Toxicity, tumour response, and LC were evaluated per treated tumour.

### Statistical analysis

Descriptive analyses were used to report characteristics of interest. Categorical variables were summarised as counts and percentages and continuous variables were presented as a median value with a range. The time from the start of MRgFUS-MB to the date of local progression was used to calculate LC on a tumour basis. LC was calculated using the Kaplan–Meier method. The R software for Windows (v2023.06.2 561 x64) was used for statistical analysis.

## Results

### Patient and tumour characteristics

**Fig 3:**
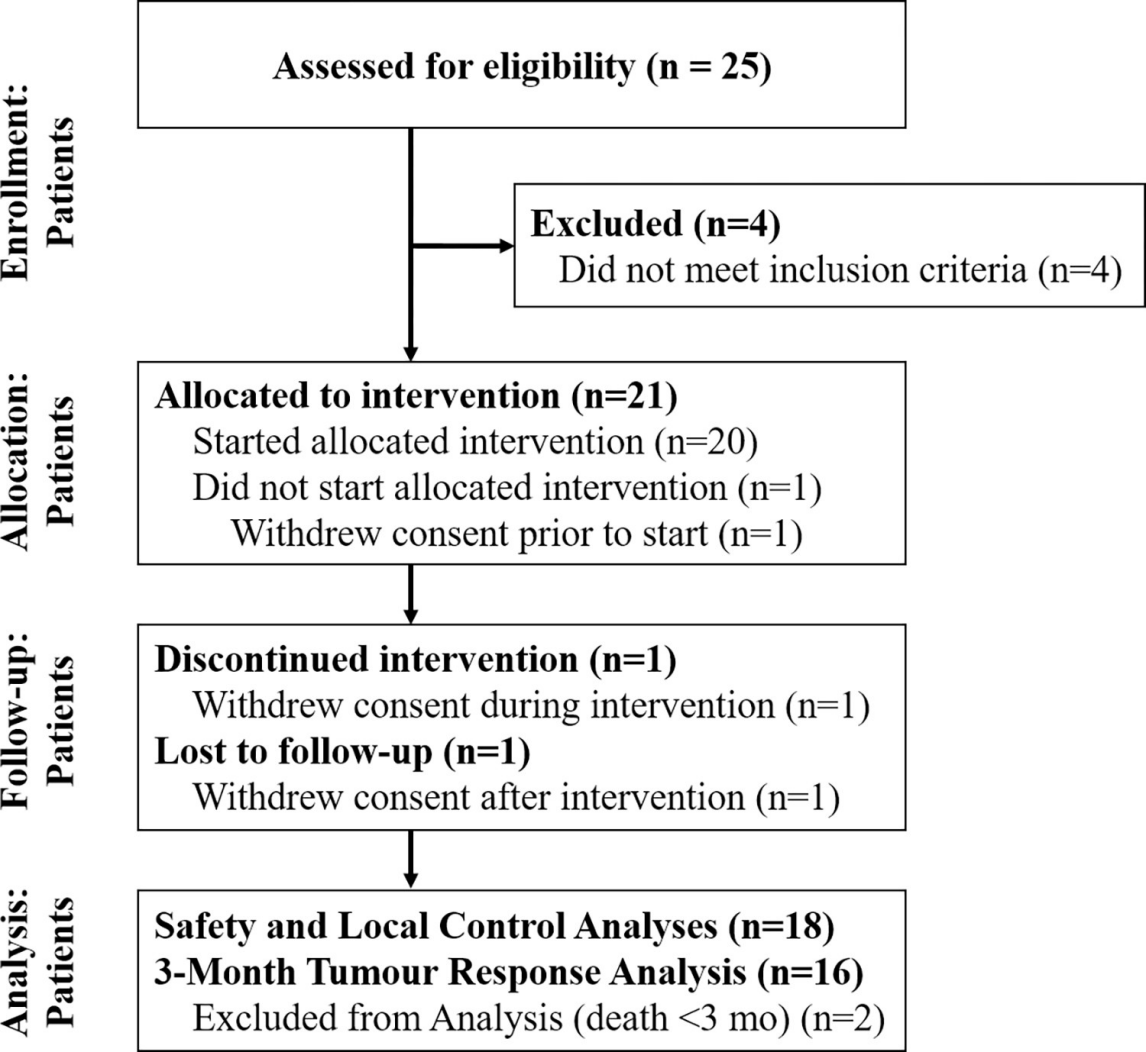
CONSORT diagram showing the flow of participants through each stage of the clinical trial. mo, months.

Between August 2020 and November 2022, a total of 21 female patients presenting with 23 primary breast tumours were enrolled and allocated to intervention (Fig 3). Among them, 3 patients subsequently withdrew consent: the first declined treatment before initiation, the second underwent the first but declined the second MRgFUS-MB treatment, and the third underwent all MRgFUS-MB treatments but withdrew consent after treatment completion, representing the only patient lost to follow-up in our study. The only toxicity reported among these 3 patients while on treatment or follow-up consisted of a single case of grade 1 radiation dermatitis. Consequently, 18 female patients with 20 primary breast tumours (median age 60 years, range 44 to 90) were included in the safety and LC analyses (Table 1). Two patients died due to progressive metastatic breast cancer (unrelated to the treated sites) before 3 months after treatment completion and were excluded from the 3-month tumour response analysis. The median follow-up was 9 months (range, 0.3 to 29). There were no protocol deviations.

**Table 1:** Patient, tumour, and treatment characteristics.

Patient /Tumour	Age (years)	TNM Stage*	Molecular subtypes	Prior breast surgery	Prior breast radiotherapy	Immediate prior systemic therapy	Treatment site	RT Dose	RT Technique	Number of MRgFUS +MB Treatments	Number of US cells	FUS power (watts)	Duration of each MRgFUS+MB treatment (min)
#1	72	T4N2M1	Basal-like	BCS	None	None	Left breast	40 Gy/10 Fx	3D-CRT	2	3	4	66 and 59
#2 Left	75	T3N2M1	HR+ HER2-	BCS	Yes, unknown details	Palbociclib, Letrozole	Right breast	30 Gy/5 Fx	3D-CRT	2	3	7	63 and 39
#2 Right	75	T3N2M1	HR+ HER2-	BCS	Yes, unknown details	Palbociclib, Letrozole	Left breast	30 Gy/5 Fx	3D-CRT	2	3	4	49 and 45
#3	59	T4N2M1	HR- HER2+	Mastectomy	None	TDM1	Right chest wall	30 Gy/5 Fx	IMRT	2	4	6	70 and 65
#4	44	T3N1M0	Basal-like	BCS	None	None	Right breast	30 Gy/10 Fx	3D-CRT	2	6	4	92 and 82
#5	59	T3N2M1	HR+ HER2-	None	None	None	Left breast	20 Gy/5Fx	3D-CRT	2	3	8	77 and 61
#6	76	T4N2M1	HR+ HER2-	BCS	50 Gy/25 Fx	Capecitabine	Left breast	20 Gy/5Fx	3D-CRT	2	5	5	96 and 88
#7	57	T4N2M1	HR+ HER2+	None	None	Trastuzumab	Left breast skin nodules	40 Gy/10 Fx	3D-CRT	2	7	4	89 and 61
#8	61	T3N2M1	Basal-like	Mastectomy	50 Gy/25 Fx	Gemcitabine, Carboplatin	Left chest wall	20 Gy/5Fx	3D-CRT	2	8	4	112 and 122
#9	51	T4N3M1	HR+ HER2-	BCS	50 Gy/25 Fx	Capecitabine	Left breast skin nodules	20 Gy/5Fx	Electrons	2	4	3	60 and 60
#10	74	T4N2M1	HR+ HER2-	None	None	Palbociclib, Letrozole	Left breast	35 Gy/5 Fx	3D-CRT	2	8	6	120 and 101
#11	67	T3N0M0	HR+ HER2-	Mastectomy	50 Gy/25 Fx; 42.6Gy/16 Fx	Fulvest, Palbociclib	Left breast skin nodules	20 Gy/5 Fx	Electrons	2	3	3	49 and 45
#12	60	T2N3M1	HR+ HER2+	Mastectomy	None	TDM1	Right chestwall	20 Gy/5 Fx	IMRT	2	5	4	78 and 62
#13	56	T3N1M1	HR- HER2+	None	None	Pertuzumab, Trastuzumab	Right breast	35 Gy/5 Fx	IMRT	2	6	4	91 and 71
#14 Left	44	T4N3M1	HR+ HER2+	None	None	Pertuzumab, Trastuzumab	Left breast	66 Gy/33 Fx	3D-CRT	3	6	4	60, 70 and 70
#14 Right	44	T4N3M1	HR+ HER2+	None	None	Pertuzumab, Trastuzumab	Right breast	66 Gy/33 Fx	3D-CRT	3	6	4	70, 70 and 60
#15	88	T2N1M1	HR+ HER2-	Lumpectomy	20 Gy/5 Fx	Letrozol	Left breast	20 Gy/5 Fx	3D-CRT	2	5	4	65 and 60
#16	63	T2N1M1	HR+ HER2—	Lumpectomy	42.6Gy/16 Fx	Anastrozole	Right chest wall	35 Gy/5 Fx	IMRT	2	2	4	45 and 34
#17	50	T3N1M1	HR+ HER2-	None	25 Gy/15 Fx	Letrozole, Ribociclib.	Left breast	20 Gy/5 Fx	3D-CRT	2	8	5	90 and 90
#18	90	T2N1M1	HR+ HER2-	None	None	Letrozol	Right breast	35 Gy/5 Fx	IMRT	2	7	5	95 and 100

* TNM Stage per the 8th Edition of the American Joint Committee on Cancer (AJCC) Cancer Staging Manual [25].

BCS, breast-conserving surgery; 3D-CRT, three-dimensional conformal radiotherapy; IMRT, intensity-modulated radiotherapy; HR, hormone-receptor; HER2, human epidermal growth factor receptor 2; MRgFUS-MB, magnetic resonance-guided focussed ultrasound-stimulated microbubble; FUS, focused ultrasound; min, minutes.

All but 2 patients had metastatic disease. When analysing characteristics per tumour, the molecular subtypes consisted of hormone receptor-positive human epidermal growth factor receptor 2 (HER-2)-negative in 55% (*n* = 11/20), HER-2-positive in 30% (*n* = 6/20), and triple-negative in 15% (*n* = 3/20). Eighty percent (*n* = 16/20) of the target tumours were localised in the breast and 20% (*n* = 4/20) were in the chest wall. Forty percent of patients (*n* = 8/20) had locoregional radiotherapy, 20% (*n* = 4/20) had whole breast radiotherapy, and 40% (*n* = 8/20) underwent focal treatment (i.e., partial breast radiotherapy). The prescribed dose was 20 Gy/5 fractions (40%, *n* = 8/20), 30 to 35 Gy/5 fractions (35%, *n* = 7/20), 30 to 40 Gy/10 fractions (15%, *n* = 3/20), and 66 Gy/33 fractions (10%, *n* = 2/20). Sixty-five percent of treatments (*n* = 13/20) were delivered with 3D radiotherapy (field-in-field beam arrangement), 25% (*n* = 5/20) with IMRT, and 10% (*n* = 2/20) using electrons. Ninety percent (*n* = 18/20) of tumours underwent 2 MRgFUS-MB treatments and 10% (*n* = 2/20) underwent 3 treatments. The median number of ultrasound treatment cells per MRgFUS-MB treatment was 5 (range, 2 to 8). The median duration of each MRgFUS-MB treatment was 70 min (range, 34 to 122).

### Safety

**Fig 4:**
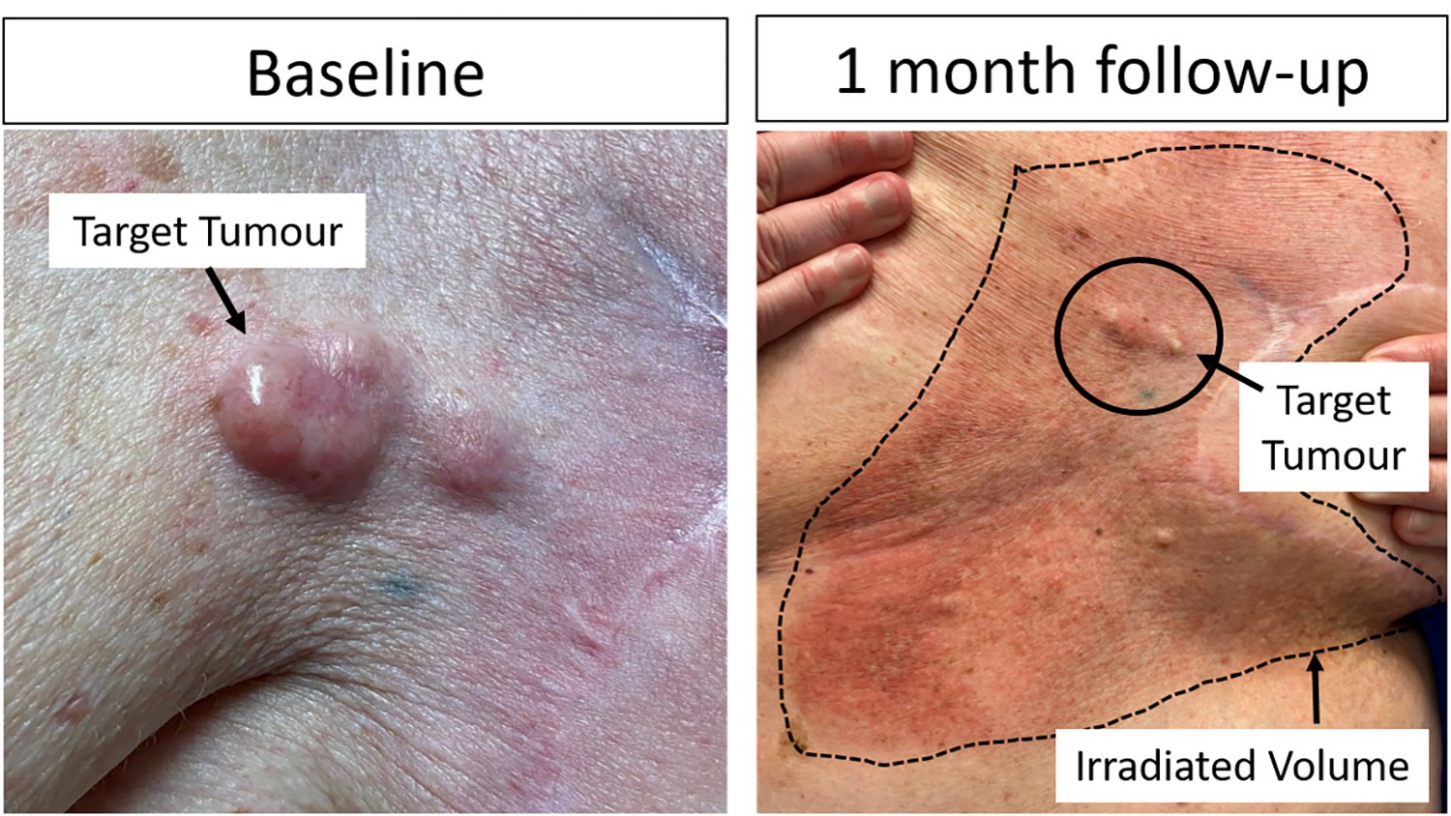
Example of radiation dermatitis following focussed ultrasound-stimulated microbubbles radiation enhancement treatment. A patient with cutaneous skin nodules (identified as patient #9 in Tables 1 and 2) received radiation therapy comprising 20 Gy in 5 fractions with electrons to the area delineated by a dotted line. The nodule, identified at baseline image and demarcated by a solid black line at the one-month follow-up, was the target for radioenhancement treatment with MRgFUS-MB. The most severe observed toxicity was grade 1 dermatitis, and the region treated with combined treatment (MRgFUS-MB + radiation) displayed a similar intensity of cutaneous toxicity compared to the areas treated with radiotherapy alone, suggesting that radiation dermatitis would likely have occurred to a comparable extent in the absence of radioenhancement therapy.

All 18 patients (20 tumours) were included in the safety analysis and were followed for a minimum of 7 days posttreatment. The most common acute adverse event was radiation dermatitis, which was categorised as grade 1 in 75% (*n* = 15/20), grade 2 in 5% (*n* = 1/20), and grade 3 in 10% of treatments (*n* = 2/20). The 3 cases of grade≥2 dermatitis occurred following whole breast radiotherapy with 35 Gy in 5 fractions (1 patient, grade 3), whole breast radiotherapy with 66 Gy in 33 fractions (1 patient, grade 3), and stereotactic body radiotherapy to a recurrent chest wall tumour with 30 Gy in 5 fractions (1 patient, grade 2). There was no difference in the severity of radiation dermatitis between target and non-target areas with MRgFUS-MB therapy (exemplified in Fig 4). One patient experienced a grade 1 allergic reaction (characterised by a mild cough) approximately 30 min after the ultrasound-stimulated microbubble treatment, which did not necessitate any intervention. While it could also be associated with the MR contrast media (gadolinium) administered after MRgFUS-MB treatment completion, no other instances of allergic reactions following contrast-enhanced MR were reported for this patient. Consequently, it was deemed probably related to the microbubble administration. No other cases of systemic complications of MRgFUS-MB or allergic reactions were reported. Furthermore, no instances of radiation necrosis were observed during the long-term follow-up.

**Table 2:** Data on acute toxicity and tumour response.

Patient/tumour	Contrast-enhancing tumour size at various time points (mm)				Acute toxicity			Tumour response	
	Baseline	1 week follow-up	1 month follow-up	3 months follow-up	Systemic adverse events	Radiation dermatitis	Other grade ≥ 2 toxicity	Tumour response at 3 months	Tumour response at last follow-up
1	59 × 48	57 × 42	48 × 27	0 × 0	None	Grade 1	None	CR/Replacement Fibrosis	No PD at 34 months
2 Left	24 × 24	26 × 21	24 × 21	15 × 15	None	Grade 1	None	CR/Replacement Fibrosis	No PD at 33 months
2 Right	27 × 23	27 × 25	23 × 24	22 × 18	None	Grade 1	None	PR	No PD at 33 months
3	23 × 15	19 × 22	17 × 14	0 × 0	None	Grade 2	None	CR/Replacement Fibrosis	No PD at 29 months
4	53 × 42	43 × 39	34 × 28	11 × 16	None	Grade 1	None	PR	**PD at 8 months**
5	38 × 28	17 × 20	16 × 14	14 × 14	None	Grade 1	None	CR/Replacement Fibrosis	No PD at 12 months
6	37 × 27	36 × 32	35 × 27	30 × 26	None	Grade 1	None	SD	**PD at 15 months**
7	62 × 45	35 × 31	23 × 24	0 × 0	None	Grade 1	None	CR/Replacement Fibrosis	No PD at 3.9 months
8	88 × 68	102 × 63	61 × 63	NA	None	Grade 1	None	NA	No PD at 1 month
9	25 × 12	29 × 8	22 × 7	28 × 16	None	Grade 1	None	**PD**	**PD at 3 months**
10	38 × 31	38 × 31	29 × 27	29 × 27	None	Grade 3	None	CR/Replacement Fibrosis	No PD at 18 months
11	24 × 5	10 × 3	0 × 0	0 × 0	None	Grade 0	None	CR/Replacement Fibrosis	No PD at 18 months
12	35 × 21	31 × 18	35 × 11	26 × 10	Possible Grade 1 Allergic Reaction	Grade 1	None	PR	No PD at 14 months
13	23× 29	28 × 21	19 × 18	18 × 12	None	Grade 1	None	PR	No PD at 12 months
14 Left	92 × 11	62 × 12	65 × 14	65 × 14	None	Grade 3	None	CR/Replacement Fibrosis	No PD at 9 months
14 Right	46 × 13	38 × 10	0 × 0	0 × 0	None	Grade 1	None	CR/Replacement Fibrosis	No PD at 9 months
15	76 × 35	63 × 15	61 × 13	58 × 15	None	Grade 1	None	PR	No PD at 12 months.
16	39 × 28	34 × 28	34 × 28	35 × 31	None	Grade 1	None	SD	No PD at 8 months
17	29 × 19	21 × 19	16 × 18	15 × 16	None	Grade 1	None	PR	No PD at 8 months
18	46 × 50	52 × 41	NA	NA	None	Grade 1	None	NA	No PD at 1 week

Patients were assigned numbers from 1 to 18, and for those with bilateral tumours, the left and right sides were described.

CR, complete response; NA, not available; PD, progressive disease; PR, partial response; SD, stable disease.

### Tumour response and local control

**Fig 5:**
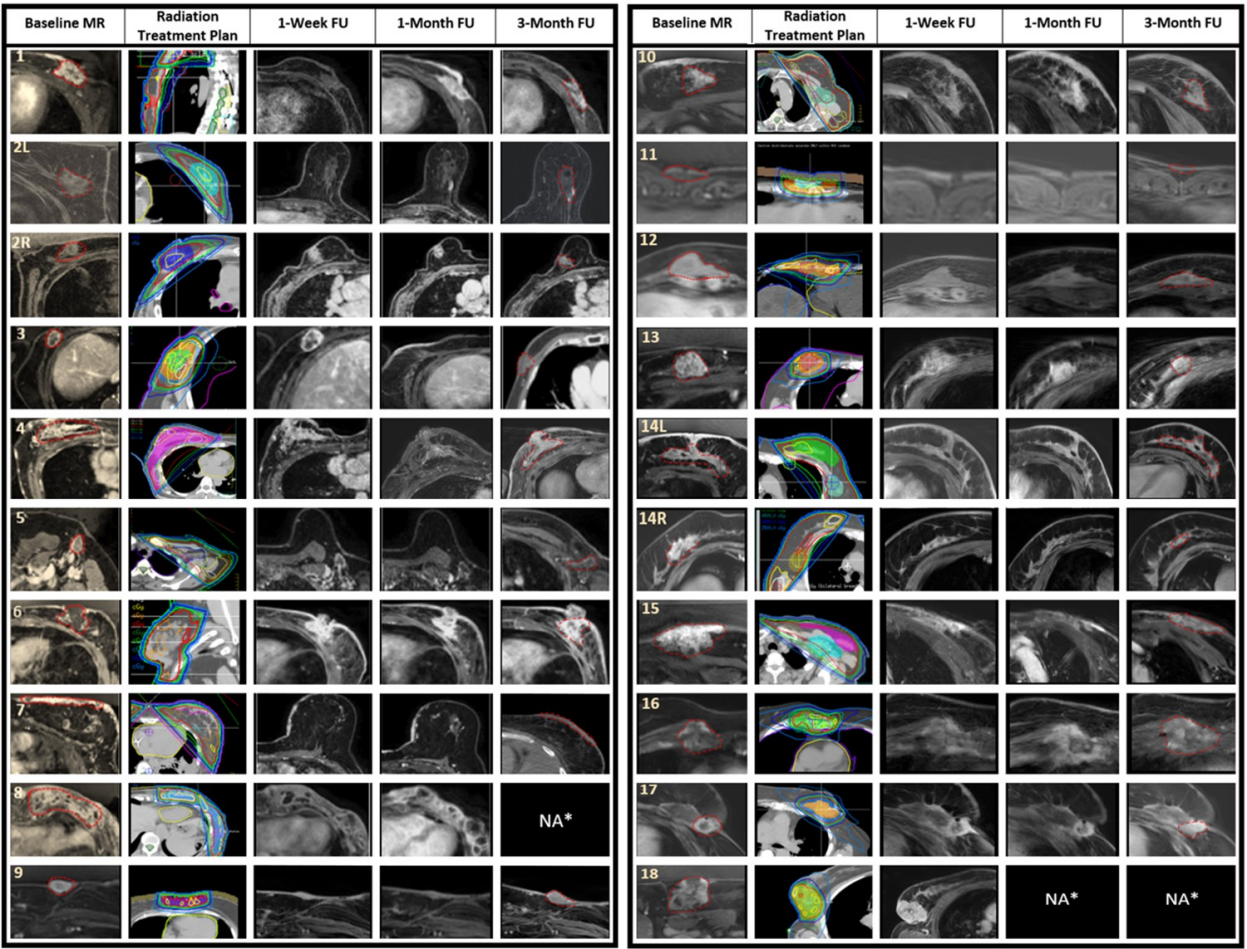
Baseline imaging and radiological follow-up at different time points. The figure details the radiation treatment plan and magnetic resonance imaging of the target tumour at baseline, 1 week, 1 month, and 3 months posttreatment. Patients were assigned numbers from 1 to 18, and for those with bilateral tumours, the left and right sides were denoted by “L” and “R,” respectively (i.e., “2L” and “2R”). The dotted red line represents the target tumour at baseline and the residual tumour or replacement fibrosis at 3 months follow-up imaging. In the radiation treatment plan, the 105% isodose line was represented in yellow, the 100% in red, the 95% in green, the 80% in dark blue, and the 50% in light blue. *Two patients died before the 3-month follow-up, represented by NA*. FU, follow-up; MR, magnetic resonance imaging; NA, not available; L, left; R, right.

**Fig 6:**
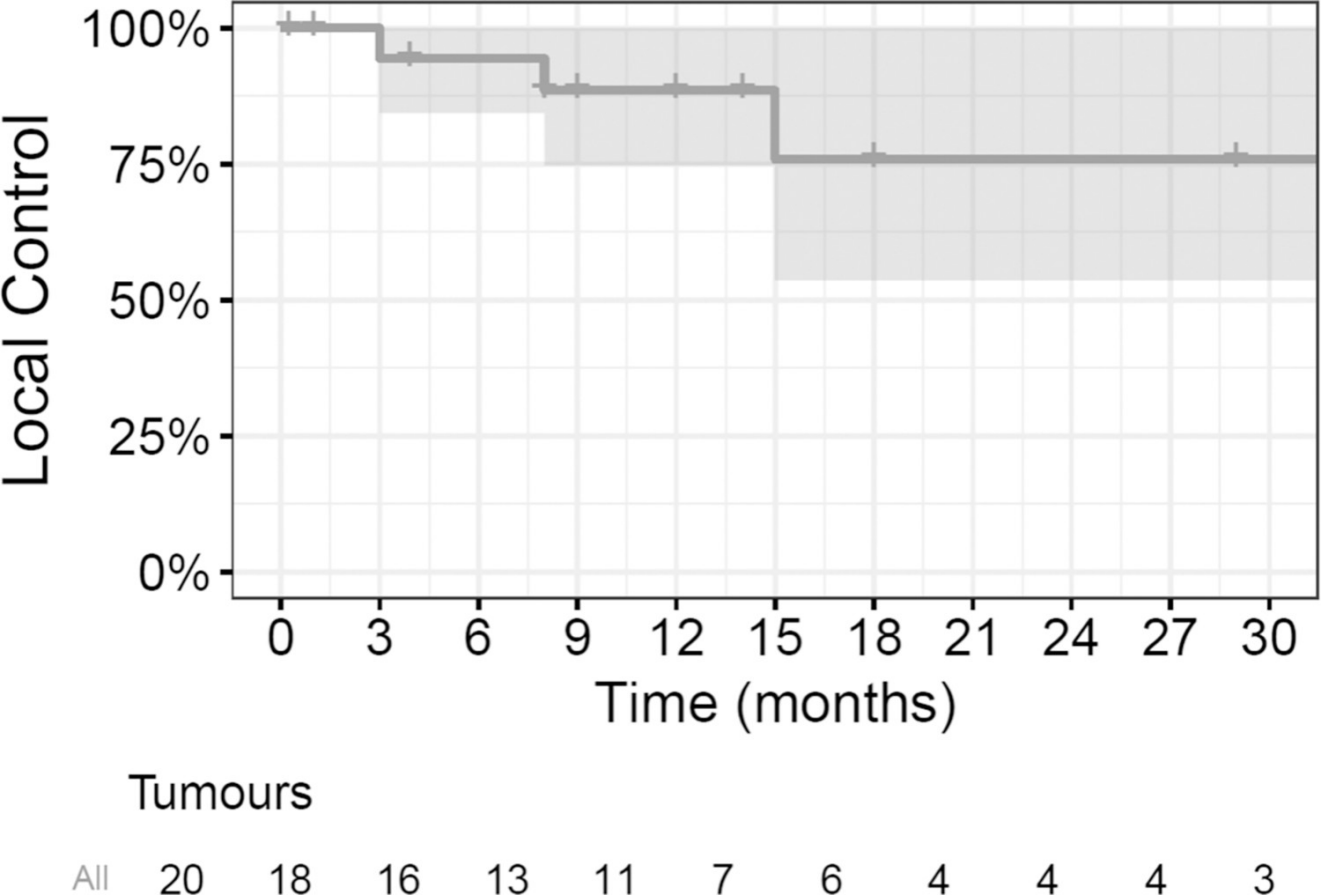
Kaplan–Meier curve for local control of the target tumours. The solid line depicts the probability of local control for the 20 target tumours; the shaded area signifies the 95% confidence interval around the estimated local control probability; each step denotes an event of local failure; a short vertical line without a drop indicates the tumour was censored.

A total of 16 patients with 18 tumours had radiological follow-up at 3 months and were assessed for tumour response. At 3 months, 50% had complete (*n* = 9/18) and 33% partial (*n* = 6/18) response, 11% had stable disease (*n* = 2/18), and 6% (*n* = 1/18) had progressive disease. Among patients with complete responses, we observed cases in which the tumour disappeared, as exemplified by tumour #14R, and cases in which it was replaced by fibrotic tissue with no evidence of contrast enhancement on MR, exemplified by tumour #5 (Table 2 and Fig 5). On subsequent follow-up, 2 patients who initially achieved stable disease (*n* = 1) and partial response ultimately presented tumour progression at 15 and 8 months, respectively (tumour #4 and #6, Table 2 and Fig 5). No patients in the study here underwent subsequent surgical resection of the target tumour. The LC rate at 6, 12, and 24 months was 94% (95% CI [84%, 100%]), 88% (95% CI [75%, 100%]), and 76% (95% CI [54%, 100%]), respectively (Fig 6). Cases of sustained LC for over 2 years were exemplified by tumours #1–3 in Table 2.

## Discussion

This study presents the mature outcomes of what is, to our knowledge, an innovative therapy aimed at enhancing radiation efficacy for breast cancer treatment. We observed a safe profile, with no grade≥3 microbubble-related adverse events. Additionally, 83% of tumours achieved partial or complete responses and the LC rate at 2 years was 76% (95% CI [54%, 100%]). Our research addresses a critical unmet need—the development of radiosensitizers capable of selectively enhancing radiation efficacy without increasing toxicity, thereby improving the therapeutic ratio. Our treatment approach is based on mechanical perturbation of the endothelial cells lining the tumour vasculature, which was shown in preclinical data to up-regulate pro-apoptotic pathways, ultimately enhancing tumour cell death [8–14,20]. To our knowledge, this is the first clinical trial to date to combine focussed ultrasound-stimulated microbubbles and external beam radiotherapy.

We observed an objective response rate of 83% at 3 months, with the majority of patients showing tumour disappearance or absence of contrast enhancement, indicating disease replacement by fibrotic changes. Furthermore, among patients with replacement fibrosis, none exhibited progressive disease on follow-up imaging. We observed potentially encouraging LC rates of 88% at 1 year and 76% at 2 years, despite the fact that most patients received palliative radiation doses, such as 20 Gy/5 fractions or 30 to 40 Gy/10 fractions. Our efficacy data are comparable to that of other studies involving patients treated with higher radiation doses. For example, Moore-Palhares and colleagues [6] reported an objective response rate of 54% (8% complete and 54% partial responses) at the last follow-up and a 2-year LC rate of 89% in the treatment of non-resected breast tumours using ablative radiotherapy doses (35 to 40 Gy in 5 fractions). Furthermore, Webb and colleagues [26] observed an objective response rate of 87% (10% complete and 77% partial responses), but a lower 2-year LC rate of 44% following treatment with 30 to 36 Gy in 5 to 6 fractions. Taken together, our results align with the radioenhancement potential of MRgFUS-MB, as supported by extensive preclinical studies [8–14,20].

Our data demonstrate the safety of this approach, with no significant microbubble-related adverse events. The only systemic adverse event probably associated with microbubble administration was a grade 1 allergic reaction, which did not require any treatment. The definity microbubbles employed in the trial here have been commercially used over several years and have consistently demonstrated a well-established safety profile [27,28]. Systemic adverse events associated with microbubble administration are uncommon and typically mild, whereas the occurrence of significant toxicity, such as anaphylactic reactions, is limited to less than 0.001% of patients [27,28]. All grades 2 and 3 adverse events observed in our study were attributed to the expected radiation dermatitis resulting from high prescribed doses of radiation (66 Gy in 33 fractions and 30 to 35 Gy in 5 fractions). Moreover, the intensity of radiation dermatitis in each case was similar between the target site and the regions not treated with MRgFUS. This suggests that the radioenhancement treatment did not lead to increased radiation dermatitis toxicity and that this toxicity would likely have occurred similarly in the absence of the experimental treatment. This finding aligns with the capability of focussed ultrasound to accurately stimulate microbubbles at the intended target site without affecting surrounding normal tissue. This selectivity is essential to enhance tumour response without increasing toxicity, with consequent improvement in the therapeutic index.

The synergy between focussed ultrasound-stimulated microbubbles and radiation therapy has also been investigated for different primary tumours. At our institution, 2 ongoing Phase 1 clinical trials are enrolling patients with non-resected or metastatic melanoma or skin cancer (ClinicalTrials.gov Identifier: NCT05620290) or locally advanced primary head and neck cancer [29] (ClinicalTrials.gov Identifier: NCT04431648) to undergo ultrasound-stimulated microbubbles in combination with standard radiation treatment, and sustained complete responses have been observed [29]. Additionally, employing a distinct approach, a Phase II clinical trial conducted at Thomas Jefferson University is randomising patients with hepatocellular carcinoma to receive transarterial radioembolization (TARE) either with or without ultrasound-stimulated microbubbles (ClinicalTrials.gov Identifier: NCT03199274). Preliminary results reported from that work are potentially encouraging and have demonstrated improved treatment response among patients treated with the combined approach [30]. Therefore, data from these clinical studies will be crucial to explore this radioenhancement treatment in different scenarios.

In order to further advance the utilisation of MRgFUS-MB treatment for breast cancer patients, the results here support a larger Phase 2 clinical trial, aiming to confirm the safety and efficacy of the present study. Upcoming work will benefit from next-generation ultrasound therapy devices capable of simultaneously stimulating microbubbles in an entire tumour over the duration of a few minutes. In contrast, the focussed ultrasound employed in the current study activates treatment cells sequentially using a step and shoot approach until the entire target has been treated. Consequently, patients experiencing claustrophobia or pain may encounter difficulties lying prone on the MR table for extended periods, even after administering anxiolytics or analgesics. This is exemplified by an elderly patient who underwent the first but declined the second MRgFUS-MB treatment and further follow-up, rendering exclusion from this current analysis. However, the next-generation equipment holds promise for a much shorter treatment duration, which is expected to enhance treatment tolerance and adherence [31].

Potential approaches for future studies could involve the combination of MRgFUS-MB and neoadjuvant stereotactic body radiotherapy, aiming to increase the pathological complete response of disease. Additionally, studies could focus on including non-operable breast cancer patients undergoing definitive radiotherapy and investigate whether the addition of MRgFUS-MB improves LC. For those with unresectable tumours, further investigation could explore whether this radioenhancement treatment could significantly downstage the tumours and increase operability. Furthermore, as there are still uncertainties regarding the optimal number and frequency of MRgFUS-MB treatments, trials comparing the safety and efficacy of different treatment schedules could provide valuable insights into the most effective approach. And lastly, an opportunity exists to integrate MRgFUS-MB therapy into MR linear accelerator machines, streamlining the administration of radioenhancement treatment immediately before radiotherapy exposure.

The trial here has several strengths. To our knowledge, this is the first clinical trial to combine ultrasound-stimulated microbubbles and external beam radiotherapy, and there is compelling prior preclinical data supporting this approach. Patients were followed closely during and after treatment to ensure safety, and tumour response was systematically assessed with regular MR imaging, which is the recommended breast imaging modality to assess response due to its ability to differentiate residual tumour from non-enhancing replacement fibrosis. However, the study here also has limitations, including variable tumour volume, treatment indications, different dose fractionation regimens, variable long-term follow-up routine, and the anticipated small sample size typical for a Phase 1 clinical trial. Furthermore, while this study primarily focuses on the theory that the up-regulation of the ASMase-ceramide pathway is the main driver of the synergistic effect with radiation, it is important to acknowledge the potential influence of other mechanisms triggered by ultrasound-stimulated microbubbles. These mechanisms could include enhanced tumour perfusion resulting in temporary improved oxygenation or the activation of alternative cell death signalling pathways unrelated to the ASMase-ceramide pathway, which could independently contribute to the radioenhancement effect [21,32,33].

In conclusion, MRgFUS-MB treatment is an innovative and potentially promising radioenhancement therapy. Despite being safe, it led to high rates of objective response and sustained LC. Our data carry substantial implications, potentially opening new avenues for improving treatment outcomes in breast cancer and possibly facilitating the replication of this approach other primary malignancies, thereby optimising cancer treatment strategies. The safety and efficacy results seen in this trial should be confirmed in large Phase 2 clinical trials.

## Supporting information

S1 Checklist Transparent Reporting of Evaluations with Nonrandomized Designs (TREND) statement checklist. (DOC)

## Abbreviations

3D-CRT: three-dimensional conformal radiotherapy
ASMase: acid sphingomyelinase
CI: confidence interval
CTCAE: Common Terminology Criteria for Adverse Events
ECOG: Eastern Cooperative Oncology Group
FUS-MB: focussed ultrasound-stimulated microbubble
HER-2: human epidermal growth factor receptor 2
IMRT: intensity-modulated radiotherapy
LC: local control
MR: magnetic resonance
MRgFUS-MB: magnetic resonance-guided focussed ultrasound-stimulated microbubble
RECIST: Response Evaluation Criteria in Solid Tumors V1.1
TARE: transarterial radioembolization
TREND: Transparent Reporting of Evaluations with Nonrandomized Designs
